# Three-dimensional entrainment using global cardiac chamber mapping

**DOI:** 10.1016/j.hrcr.2022.11.003

**Published:** 2022-11-15

**Authors:** Tamas Szili-Torok, Rita B. Gagyi, Wim Bories, Andre de Wit, Sing-Chien Yap

**Affiliations:** ∗ Erasmus Medical Center, Rotterdam, The Netherlands; † Acutus Medical Inc, Zaventem, Belgium

**Keywords:** Entrainment mapping, Reentrant arrhythmia, Global chamber mapping, Noncontact mapping, 3D mapping

**Key Teaching Points**

• Dipole charge density mapping is a unique mapping modality extending the current utility of entrainment mapping in the diagnosis and mapping of atrial tachycardias.
• This novel noncontact mapping method offers global chamber mapping of atrial arrhythmias.
• Using 3D activation pattern–based entrainment, we are able to discriminate between the critical isthmus and non-isthmus sites within the arrhythmia circuit. 3D entrainment may add a new diagnostic tool for very complex arrhythmias.

## Introduction

Our understanding of the precise mechanisms underlying atrial tachyarrhythmias is continuously evolving. A substantial portion of atrial tachyarrhythmias targeted for ablation have reentry mechanism. Entrainment mapping for reentrant arrhythmias was first described by Waldo and colleagues^1^ more than 45 years ago. Since the implementation, entrainment mapping has become a powerful tool in everyday practice.^2^ In the last decades, enormous progress has been made in electroanatomic mapping technology. Recently, the novel dipole charge density-based high-resolution mapping system (AcQMap; Acutus Medical, Carlsbad, CA) was introduced that can provide accurate characterization of atrial tachyarrhythmia mechanisms on a real-time echocardiography-based anatomy reconstruction.^3^ The AcQMap displays waves of electrical activation across the 3-dimensional (3D) anatomy reconstruction through time as high-resolution propagation history maps. More importantly, it provides single-beat high-resolution maps. Based on this we hypothesized that combining high-resolution propagation and single-beat maps can visualize 3D activation patterns during classical electrophysiology maneuvers such as entrainment mapping.

The aim of this first-in-human proof-of-concept paper was to demonstrate a novel 3D-enhanced entrainment technique using global chamber mapping.

## Case report

A 51-year-old male patient presented with recurrent episodes of palpitations. He was first diagnosed with supraventricular tachycardia, his electrocardiogram showing a narrow complex tachycardia in 2011. He presented with recurring symptoms every following year from 2011 to 2018; cardioversion was performed with adenosine administration in all cases. In 2018 the patient eventually underwent successful catheter ablation of a left-sided concealed accessory pathway. After 24 months the patient returned with highly symptomatic palpitations differing from his previous arrhythmia and his electrocardiogram was suggestive for typical atrial flutter.

In March 2022 the patient underwent an electrophysiology (EP) study again. His EP study excluded the recurrence of his concealed bypass tract and the findings were consistent with typical counterclockwise atrial flutter that was mapped using the AcQMap system. After positioning of the decapolar catheter in the coronary sinus (CS) and heparin administration for an ideal activated clotting time (>300 s), the basket catheter was introduced in the right atrium (RA) through a 12.4F AcQGuide sheath. Anatomical reconstructions of the RA were created by positioning the basket catheter in the middle of the chamber. Each one of the 6 splines of the basket catheter contains 8 ultrasound transducers, which collect up to 115,000 ultrasound data points per minute for anatomy reconstruction. The 8 high-fidelity low-impedance electrodes on every spline of the basket catheter collect up to 150,000 biopotential samples per second, which are used to create high-resolution propagation history maps of electrical activation.

**Figure 1:**
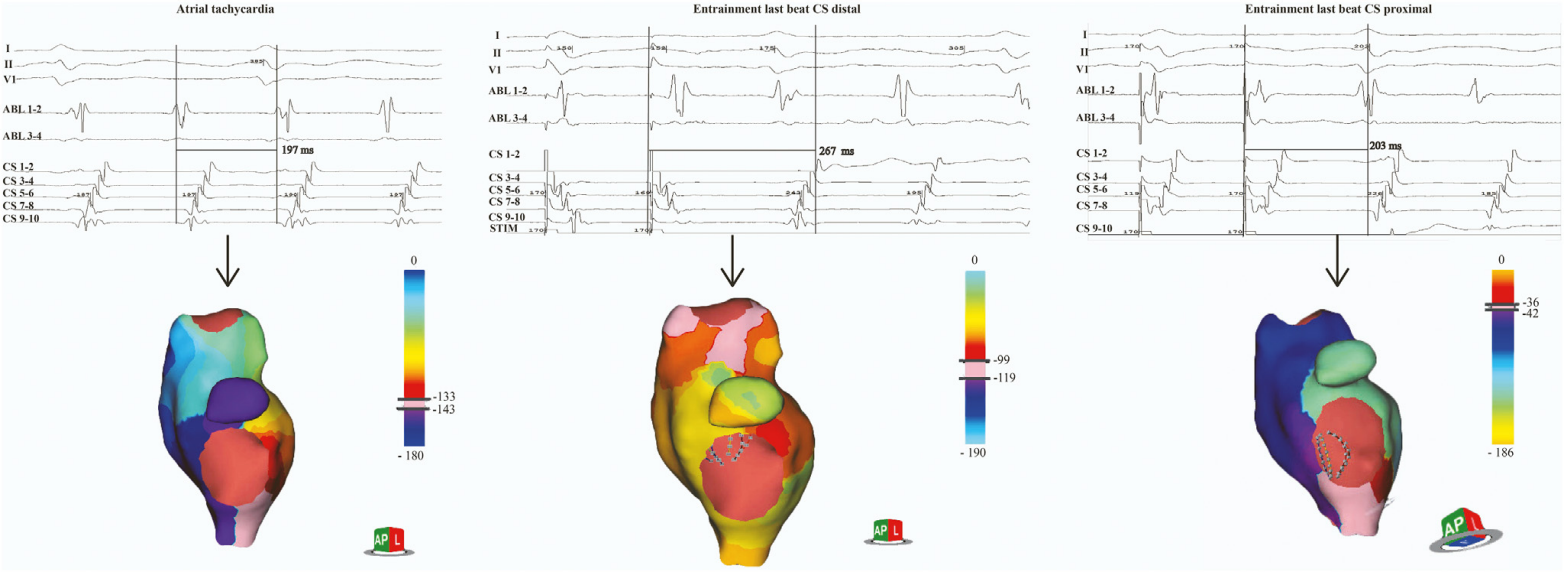
The concept of 3-dimensional entrainment. The originally mapped tachycardia had a tachycardia cycle length (TCL) of 197 ms (left). Entrainment pacing from the distal coronary sinus (CS 1-2) resulted in a postpacing interval (PPI) of 267 ms (PPI−TCL +70 ms) and a completely different propagation history map of electrical activation (middle). During entrainment pacing from the proximal CS we documented a PPI of 203 ms (PPI−TCL 6 ms) and quasi-identical propagation history map with the original tachycardia (right). Color scales are shown for every propagation map separately. For every color scale first the cycle length is introduced, then the interval is selected (the smaller the interval, the more detailed the propagation map). When color bands are close to each other a slower conduction can be identified.

On atrial burst pacing atrial flutter was inducible. Single-position maps and aggregated multiposition maps of electrical activation were achieved during ongoing counterclockwise cavotricuspid isthmus (CTI)-dependent flutter. We repeated 3D dipole charge density maps during entrainment mapping from the CS and from the CTI, respectively. The tachycardia cycle length (TCL) was 197 ms. The activation pattern on the diagnostic CS catheter showed the earliest atrial signal in the proximal CS (CS 9-10). Entrainment pacing from the distal CS (CS 1-2) at TCL-20 ms resulted in a postpacing interval (PPI) of 267 ms (PPI−TCL +70 ms) and a completely different propagation history map of electrical activation (manifest entrainment) compared to the originally mapped tachycardia (Supplemental Video 1). Only measurements with reproducible PPI were included. During entrainment pacing from the proximal CS at TCL minus 20 ms we documented a PPI of 203 ms (PPI-TCL 6 ms, Supplemental Video 2) and quasi-identical propagation history map with the original tachycardia (concealed entrainment, Supplemental Video 3; Figure 1).

Ablation was performed using an AlCath Flutter Flux Black G ablation catheter (Biotronik, Berlin, Germany), starting with a CTI line ablation from the ventricular side with the following power settings: 40 W power, 30 mL/min flow, 43°C temperature. After the second radiofrequency application the arrhythmia terminated. Single-position and aggregated multiposition maps were then repeated while applying pacing maneuvers from CS 9-10. RA activation showed bidirectional CTI block.

## Discussion

In this case presentation we present a novel concept that has the potential to change the way in which one of the most important mapping tools is used currently in cardiac electrophysiology and mapping of tachyarrhythmias. By applying 3D entrainment, we can continuously acquire and combine propagation history maps generated by the AcQMap system during entrainment mapping in a series of patients with an ongoing tachycardia. We presumed that stimulating at different sites in the atrium during the ongoing arrhythmia will change the propagation pattern accordingly. The magnitude of the pattern similarity should be depending on the distance from the exit point from the critical isthmus. Theoretically, pacing at the exit or very adjacent to the exit point from the critical isthmus should provide a quasi-identical 3D propagation map to the actual tachycardia propagation maps (concealed entrainment). At that spot classical “concealed” entrainment confirmed our hypothesis and obviously PPI is very close to the TCL. The diagnosis of concealed entrainment can only be made after manifest entrainment has been demonstrated at another site to prove the existence of a reentrant circuit with an excitable gap.

Using 3D entrainment, we can easily discriminate between manifest and concealed entrainment. When we perform classical entrainment mapping with the presence of multipolar catheters, manifest and concealed entrainment could be well discriminated. However, when a single catheter such as the ablation catheter is solely used in the atria, these abovementioned entities remain undetermined, making the interpretation very challenging. Obviously, in the ventricles the QRS morphology drives the identification of manifest entrainment vs concealed entrainment in most cases. Using our hypothesis, it opens new perspectives to achieve this on the atrial level, even having a single beat mapped after discontinuation of the entrainment pacing.

To demonstrate the feasibility of the above-described concept, we selected a patient with typical counterclockwise atrial flutter. Because our paper is proof-of-concept in nature, we opted for the best-understood mechanism of a macroreentry tachycardia to make sure that diagnostic challenges will not interfere with our measurements and mapping. With this case we could clearly show that activation maps acquired during entrainment mapping correspond to our primary hypothesis (Figure 1).

Another aspect of this method is that it can be used in a more universal way. One of the most important advantages of dipole charge density mapping is that it discriminates between reentry and focal mechanism. In nonentrainable tachycardias with focal characteristics where the PPI is rather meaningless, the pacing maneuvers are still valid and more comparable to a sort of 3D intracardiac pace mapping.

Another important advantage of this technique is that theoretically 2 adjacent beats are enough to perform this mapping. By analyzing the global activation pattern differences (or identifying the level of similarity) between the last paced and the first nonpaced beat, mapping should identify whether the catheter is located on the isthmus of the tachycardia.

Although it is beyond the current stage of the development, we aim to present the concept that using 3D activation pattern–based entrainment will discriminate between the critical isthmus (especially the exit) and non-isthmus sites within the flutter circuit. Although the PPI will be good in the latter, it will more distort the 3D activation pattern of the chamber owing to the larger extent of antidromic wavefront penetration than pacing on the critical isthmus. Entrainment from the isthmus exit will always give concealed entrainment because antidromic penetration is confined within the isthmus, in contrast to the isthmus entry.

Although our case demonstration suggests that our theory and hypothesis is valid, it was tested in only 1 simple case. It requires further studies to extend its use for more complex arrhythmias or even implementation in the ventricles. Despite this, the first experience is very promising and has the potential to elevate entrainment mapping to a completely different level. Another issue is obviously its potential cost-benefit ratio. Classical entrainment mapping is a simple pacing maneuver followed by interval measurements. It can be done in the most basically equipped EP lab. Although its use is beneficial, it has important limitations too. Our technique requires global chamber mapping. This is usually associated with extra costs, and the number of available systems is very limited. On the other hand, once it is available it can add tremendous extra information about the mechanism and propagation pattern of the atria. With this new possible feature, it may add a new diagnostic tool for very complex arrhythmias for the system is planned to be used.

## Conclusion

In conclusion, we present a novel mapping modality extending the current utility of entrainment mapping in the diagnosis and mapping of atrial tachycardias.

## Appendix Supplementary Data

Supplemental Video 1 Distal entrainment. The biggest difference between the CTI entrainment and the CS distal entrainment is that as the circuit enters the septum again, the posterior wall gets already activated from the CS distal pacing, resetting the lateral wall faster (manifest entrainment).

Supplemental Video 2 Proximal entrainment. Shows quasi the same activation pattern as the clinical (original) tachycardia (see Video 3).

Supplemental Video 3 Original tachycardia. CTI entrainment with concealed entrainment. Activation pattern showing a counterclockwise (CCW) typical flutter, including the pathway over the ridge between right atrial appendage (RAA) and superior vena cava (SVC).
