# Supplementary material for: Three-dimensional entrainment using global cardiac chamber mapping
Source: HeartRhythm Case Rep. 2022 Nov 15;9(2):84–6. doi: 10.1016/j.hrcr.2022.11.003 (PMC9968915; doi:10.1016/j.hrcr.2022.11.003)
Supplement: Supplementary file [file mmc4.docx]

Video 1 – Distal entrainment. The biggest difference between the CTI entrainment and the CS distal entrainment is that as the circuit enters the septum again, the posterior wall gets already activated from the CS distal pacing, resetting the lateral wall faster (manifest entrainment).

Video 2 – Proximal entrainment. Shows quasi the same activation pattern as the clinical (original) tachycardia (see Video 3).

Video 3 – Original tachycardia. CTI entrainment with concealed entrainment. Activation pattern showing a counterclockwise (CCW) typical flutter, including the pathway over the ridge between right atrial appendage (RAA) and superior vena cava (SVC).
